# 1-Benzyl-2-dimethyl­amino-3-methyl-3,4,5,6-tetra­hydro­pyrimidin-1-ium bromide

**DOI:** 10.1107/S1600536812029224

**Published:** 2012-07-04

**Authors:** Ioannis Tiritiris, Willi Kantlehner

**Affiliations:** a Institut für Organische Chemie, Universität Stuttgart, Pfaffenwaldring 55, 70569 Stuttgart, Germany; b Fakultät Chemie/Organische Chemie, Hochschule Aalen, Beethovenstrasse 1, D-73430 Aalen, Germany

## Abstract

In the title molecular salt, C_14_H_22_N_3_^+^·Br^−^, the ring incorporating the guanidinium grouping exhibits a half-chair conformation and the dihedral angle between the N—C—N and C—C—C planes is 55.0 (3)°. The C—N bond lengths in the central CN_3_ unit are 1.333 (4), 1.338 (3) and 1.341 (4) Å, indicating partial double-bond character. The central C atom is bonded to the three N atoms in a nearly ideal trigonal–planar geometry and the positive charge is delocalized in the CN_3_ plane. The distances between the N atom and the terminal methyl C atoms [1.453 (4)–1.461 (4) Å] are all close to a typical single C—N bond length.

## Related literature

For the crystal structure of *N*,*N*,*N′*,*N′*- tetra­methyl­chloro­formamidinium chloride, see: Tiritiris & Kantlehner (2008 ▶). For the synthesis of 1-methyl-2-dimethyl­amino-1,4,5,6-tetra­hydro­pyrimidine and derived guanidinium salts, see: Tiritiris & Kantlehner (2012*b* ▶). For the structure of 2-dimethyl­amino-1-(2-eth­oxy-2-oxoeth­yl)-3-methyl-3,4,5,6-tetra­hydro­pyri­midin-1-ium tetra­phenyl­borate see: Tiritiris & Kantlehner (2012*a* ▶). 
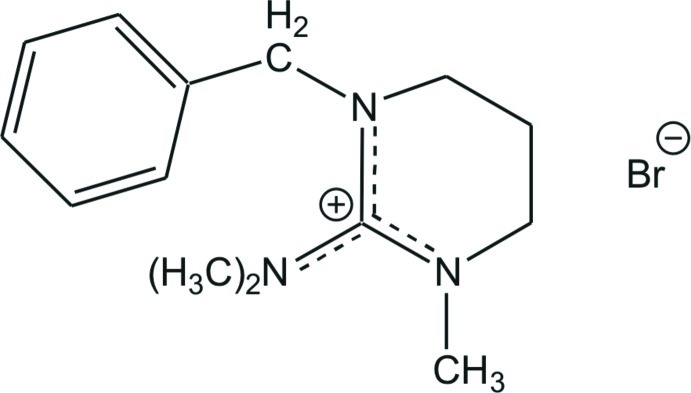


## Experimental

### 

#### Crystal data

C_14_H_22_N_3_^+^·Br^−^
*M* *_r_* = 312.25
Monoclinic, 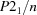

*a* = 10.7814 (7) Å
*b* = 11.8538 (8) Å
*c* = 11.4782 (8) Å
β = 93.332 (8)°
*V* = 1464.44 (17) Å^3^
*Z* = 4
Mo *K*α radiation
μ = 2.80 mm^−1^
*T* = 293 K
0.24 × 0.17 × 0.13 mm

#### Data collection

Bruker–Nonius KappaCCD diffractometer
Absorption correction: multi-scan (Blessing, 1995 ▶) *T*_min_ = 0.552, *T*_max_ = 0.695
14052 measured reflections
3536 independent reflections
1812 reflections with *I* > 2σ(*I*)
*R*_int_ = 0.064

#### Refinement

*R*[*F* ^2^ > 2σ(*F* ^2^)] = 0.035
*wR*(*F* ^2^) = 0.089
*S* = 0.81
3536 reflections
166 parameters
H-atom parameters constrained
Δρ_max_ = 0.34 e Å^−3^
Δρ_min_ = −0.49 e Å^−3^

### 

Data collection: *COLLECT* (Hooft, 2004 ▶); cell refinement: *SCALEPACK* (Otwinowski & Minor, 1997 ▶); data reduction: *SCALEPACK*; program(s) used to solve structure: *SHELXS97* (Sheldrick, 2008 ▶); program(s) used to refine structure: *SHELXL97* (Sheldrick, 2008 ▶); molecular graphics: *DIAMOND* (Brandenburg & Putz, 2005 ▶); software used to prepare material for publication: *SHELXL97*.

## Supplementary Material

Crystal structure: contains datablock(s) I, global. DOI: 10.1107/S1600536812029224/fj2572sup1.cif

Structure factors: contains datablock(s) I. DOI: 10.1107/S1600536812029224/fj2572Isup2.hkl

Supplementary material file. DOI: 10.1107/S1600536812029224/fj2572Isup3.cml

Additional supplementary materials:  crystallographic information; 3D view; checkCIF report

## Figures and Tables

**Fig. 1.**
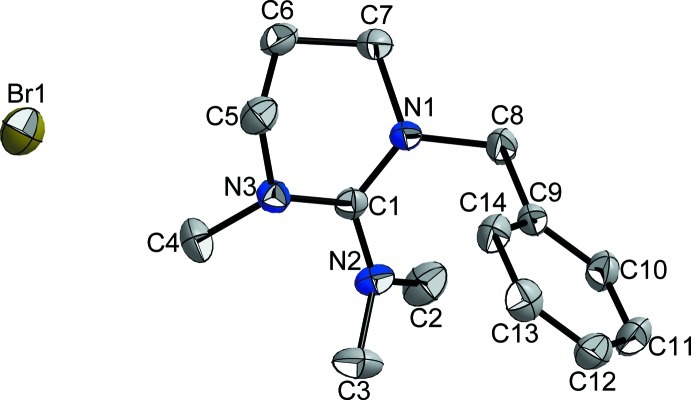
The structure of the title compound with atom labels and 50% probability displacement ellipsoids. All hydrogen atoms were omitted for clarity.

## supplementary crystallographic information

### Comment

Since we have established a simple method for synthesizing the cyclic guanidine 1-methyl-2-dimethylamino-1,4,5,6-tetrahydropyrimidine (Tiritiris & Kantlehner, 2013) from *N*,*N*,*N'*,*N'*-tetramethylchloroformamidinium chloride (Tiritiris & Kantlehner, 2008) and *N*-methyl-propane-1,3-diamine, the synthesis and characterization of related ionic tetrahydropyrimidinium derivatives, which are potentially pharmacologically active, was an aim of our investigations. The reaction of the free guanidine base with ethyl bromoacetate has been recently described by us and the resulting bromide was converted by anion exchange to the tetraphenylborate salt giving single crystals suitable for X-ray structure analysis (Tiritiris & Kantlehner, 2012). By alkylation of the free nitrogen position of the molecule with alkyl halides, it is possible to obtain guanidinium salts with a different substitution pattern, which one representative is the here presented title compound. According to the structure analysis, isolated guanidinium ions and bromide ions are present and no specific interactions between them have been observed. Prominent bond parameters in the guanidinium ion are: C1–N1 = 1.333 (4) Å, C1–N2 = 1.341 (4) Å and C1–N3 = 1.338 (3) Å. The N–C1–N angles are: 121.2 (3)° (N1–C1–N2), 119.8 (3)° (N2–C1–N3) and 118.9 (3)° (N1–C1–N3), which indicates a nearly ideal trigonal-planar surrounding of the carbon centre by the nitrogen atoms. The positive charge is completely delocalized on the CN_3_ plane. The bonds between the N atoms and the terminal *C*-methyl groups, all have values close to a typical single bond (1.453 (4)–1.461 (4) Å). All remaining C–N distances are between 1.464 (4) and 1.476 (3) Å. The six membered heterocycle exhibits a half-chair conformation (Fig. 1). The carbon atom C6 is not in the ring plane, the angle between the planes N3/C1/N1 and C5/C6/C7 is 55.0 (3)°. This value is slightly larger compared with that one determined for the guanidinium ion in 2-dimethylamino-1- (2-ethoxy-2-oxoethyl)-3-methyl-3,4,5,6-tetrahydropyrimidin-1-ium tetraphenylborate (Tiritiris & Kantlehner, 2012). The dihedral angle between the planes C1/N1/C7 and C10/C9/C14 is 66.5 (3)°, which shows a significant twisting of the phenyl ring relative to the tetrahydropyrimidine ring.

### Experimental

The title compound has been obtained by reacting equimolar amounts of 1-methyl-2-dimethylamino-1,4,5,6-tetrahydropyrimidine and benzyl bromide in acetonitrile at room temperature for two hours. After evaporation of the solvent the crude 2-dimethylamino-1-benzyl-3-methyl-3,4,5,6- tetrahydropyrimidin-1-ium bromide was washed with diethylether and dried *in vacuo*. Single crystals have been obtained by recrystallization from a saturated acetonitrile solution.

### Refinement

The hydrogen atoms of the methyl groups were allowed to rotate with a fixed angle around the C–N bond to best fit the experimental electron density, with *U*(H) set to 1.5 *U*_eq_(C) and d(C—H) = 0.96 Å. The remaining H atoms were placed in calculated positions with d(C—H) = 0.97 Å (H atoms in CH_2_ groups) and (C—H) = 0.93 Å (H atoms in the aromatic ring). They were included in the refinement in the riding model approximation, with *U*(H) set to 1.2 *U*_eq_(C).

### Crystal data

**Table:**

C_14_H_22_N_3_^+^·Br^−^	*F*(000) = 648
*M**_r_* = 312.25	*D*_x_ = 1.416 Mg m^−^^3^
Monoclinic, *P*2_1_/*n*	Melting point: 402 K
Hall symbol: -P 2yn	Mo *K*α radiation, λ = 0.71073 Å
*a* = 10.7814 (7) Å	Cell parameters from 3536 reflections
*b* = 11.8538 (8) Å	θ = 2.5–28.1°
*c* = 11.4782 (8) Å	µ = 2.80 mm^−^^1^
β = 93.332 (8)°	*T* = 293 K
*V* = 1464.44 (17) Å^3^	Block, colorless
*Z* = 4	0.24 × 0.17 × 0.13 mm

### Data collection

**Table:**

Bruker–Nonius KappaCCD diffractometer	3536 independent reflections
Radiation source: sealed tube	1812 reflections with *I* > 2σ(*I*)
Graphite monochromator	*R*_int_ = 0.064
φ scans, and ω scans	θ_max_ = 28.1°, θ_min_ = 2.5°
Absorption correction: multi-scan (Blessing, 1995)	*h* = −14→14
*T*_min_ = 0.552, *T*_max_ = 0.695	*k* = −15→15
14052 measured reflections	*l* = −15→15

### Refinement

**Table:**

Refinement on *F*^2^	Primary atom site location: structure-invariant direct methods
Least-squares matrix: full	Secondary atom site location: difference Fourier map
*R*[*F*^2^ > 2σ(*F*^2^)] = 0.035	Hydrogen site location: difference Fourier map
*wR*(*F*^2^) = 0.089	H-atom parameters constrained
*S* = 0.81	*w* = 1/[σ^2^(*F*_o_^2^) + (0.0441*P*)^2^] where *P* = (*F*_o_^2^ + 2*F*_c_^2^)/3
3536 reflections	(Δ/σ)_max_ < 0.001
166 parameters	Δρ_max_ = 0.34 e Å^−^^3^
0 restraints	Δρ_min_ = −0.49 e Å^−^^3^

### Special details

**Table:**

Geometry. All e.s.d.'s (except the e.s.d. in the dihedral angle between two l.s. planes) are estimated using the full covariance matrix. The cell e.s.d.'s are taken into account individually in the estimation of e.s.d.'s in distances, angles and torsion angles; correlations between e.s.d.'s in cell parameters are only used when they are defined by crystal symmetry. An approximate (isotropic) treatment of cell e.s.d.'s is used for estimating e.s.d.'s involving l.s. planes.
Refinement. Refinement of *F*^2^ against ALL reflections. The weighted *R*-factor *wR* and goodness of fit *S* are based on *F*^2^, conventional *R*-factors *R* are based on *F*, with *F* set to zero for negative *F*^2^. The threshold expression of *F*^2^ > σ(*F*^2^) is used only for calculating *R*-factors(gt) *etc*. and is not relevant to the choice of reflections for refinement. *R*-factors based on *F*^2^ are statistically about twice as large as those based on *F*, and *R*- factors based on ALL data will be even larger.

### Fractional atomic coordinates and isotropic or equivalent isotropic displacement parameters (Å^2^)

**Table:**

	*x*	*y*	*z*	*U*_iso_*/*U*_eq_	
Br1	0.19725 (3)	0.75077 (3)	0.59957 (2)	0.05182 (11)	
N1	0.1492 (2)	0.20807 (19)	0.58797 (18)	0.0347 (5)	
N2	0.3599 (2)	0.2078 (2)	0.5530 (2)	0.0391 (6)	
N3	0.2549 (2)	0.37602 (18)	0.57696 (19)	0.0354 (5)	
C1	0.2552 (3)	0.2632 (2)	0.5746 (2)	0.0318 (6)	
C2	0.3930 (4)	0.1018 (3)	0.6102 (3)	0.0572 (10)	
H2A	0.3435	0.0914	0.6762	0.086*	
H2B	0.4794	0.1030	0.6359	0.086*	
H2C	0.3781	0.0407	0.5562	0.086*	
C3	0.4411 (3)	0.2456 (3)	0.4638 (3)	0.0568 (8)	
H3A	0.4046	0.3098	0.4239	0.085*	
H3B	0.4517	0.1858	0.4090	0.085*	
H3C	0.5205	0.2663	0.4998	0.085*	
C4	0.3625 (3)	0.4382 (3)	0.6263 (3)	0.0514 (9)	
H4A	0.4187	0.3869	0.6668	0.077*	
H4B	0.3356	0.4941	0.6799	0.077*	
H4C	0.4039	0.4745	0.5647	0.077*	
C5	0.1329 (3)	0.4295 (2)	0.5823 (3)	0.0454 (8)	
H5A	0.0836	0.4167	0.5101	0.054*	
H5B	0.1424	0.5102	0.5937	0.054*	
C6	0.0696 (3)	0.3774 (3)	0.6841 (3)	0.0459 (8)	
H6A	0.1211	0.3866	0.7556	0.055*	
H6B	−0.0096	0.4141	0.6938	0.055*	
C7	0.0500 (3)	0.2536 (3)	0.6579 (2)	0.0431 (6)	
H7A	0.0485	0.2120	0.7306	0.052*	
H7B	−0.0297	0.2433	0.6156	0.052*	
C8	0.1207 (3)	0.0990 (2)	0.5305 (3)	0.0468 (8)	
H8A	0.0320	0.0857	0.5304	0.056*	
H8B	0.1620	0.0393	0.5757	0.056*	
C9	0.1602 (3)	0.0927 (2)	0.4063 (2)	0.0379 (7)	
C10	0.2077 (3)	−0.0062 (2)	0.3643 (3)	0.0461 (8)	
H10	0.2187	−0.0681	0.4136	0.055*	
C11	0.2395 (4)	−0.0142 (3)	0.2485 (3)	0.0548 (9)	
H11	0.2696	−0.0818	0.2203	0.066*	
C12	0.2262 (3)	0.0771 (3)	0.1770 (3)	0.0499 (8)	
H12	0.2500	0.0725	0.1005	0.060*	
C13	0.1780 (3)	0.1759 (3)	0.2172 (2)	0.0476 (8)	
H13	0.1687	0.2378	0.1677	0.057*	
C14	0.1432 (3)	0.1838 (3)	0.3311 (2)	0.0438 (7)	
H14	0.1084	0.2502	0.3573	0.053*	

### Atomic displacement parameters (Å^2^)

**Table:**

	*U*^11^	*U*^22^	*U*^33^	*U*^12^	*U*^13^	*U*^23^
Br1	0.0600 (2)	0.05218 (17)	0.04289 (15)	0.00288 (19)	−0.00030 (12)	−0.00128 (17)
N1	0.0360 (15)	0.0377 (11)	0.0313 (11)	−0.0062 (10)	0.0099 (10)	−0.0045 (9)
N2	0.0390 (16)	0.0448 (12)	0.0343 (12)	0.0072 (11)	0.0088 (10)	0.0030 (10)
N3	0.0341 (14)	0.0334 (11)	0.0388 (12)	−0.0005 (11)	0.0021 (10)	0.0032 (10)
C1	0.0370 (16)	0.0352 (14)	0.0235 (10)	−0.0006 (14)	0.0039 (10)	0.0005 (12)
C2	0.070 (3)	0.0494 (19)	0.0523 (19)	0.0201 (18)	0.0041 (17)	0.0071 (15)
C3	0.0417 (18)	0.084 (2)	0.0465 (15)	0.001 (2)	0.0164 (13)	0.005 (2)
C4	0.050 (2)	0.0432 (18)	0.060 (2)	−0.0141 (15)	−0.0044 (16)	−0.0041 (15)
C5	0.049 (2)	0.0398 (16)	0.0476 (17)	0.0102 (14)	0.0023 (15)	0.0022 (13)
C6	0.040 (2)	0.0494 (17)	0.0488 (17)	0.0084 (14)	0.0080 (14)	−0.0109 (14)
C7	0.0351 (15)	0.0513 (15)	0.0440 (13)	−0.0046 (18)	0.0115 (11)	−0.0070 (17)
C8	0.060 (2)	0.0419 (16)	0.0397 (16)	−0.0175 (15)	0.0171 (15)	−0.0114 (13)
C9	0.0402 (19)	0.0373 (14)	0.0369 (14)	−0.0109 (13)	0.0086 (12)	−0.0076 (12)
C10	0.059 (2)	0.0329 (14)	0.0465 (17)	−0.0021 (14)	0.0074 (15)	−0.0029 (12)
C11	0.068 (3)	0.047 (2)	0.0509 (17)	0.0033 (17)	0.0159 (16)	−0.0164 (16)
C12	0.052 (2)	0.064 (2)	0.0343 (15)	−0.0064 (17)	0.0079 (14)	−0.0130 (15)
C13	0.054 (2)	0.0525 (19)	0.0350 (15)	−0.0053 (16)	−0.0051 (14)	0.0023 (13)
C14	0.049 (2)	0.0402 (17)	0.0417 (16)	0.0005 (14)	−0.0004 (14)	−0.0076 (13)

### Geometric parameters (Å, º)

**Table:**

N1—C1	1.333 (4)	C5—H5B	0.9700
N1—C8	1.476 (3)	C6—C7	1.511 (4)
N1—C7	1.476 (3)	C6—H6A	0.9700
N2—C1	1.341 (4)	C6—H6B	0.9700
N2—C2	1.453 (4)	C7—H7A	0.9700
N2—C3	1.456 (4)	C7—H7B	0.9700
N3—C1	1.338 (3)	C8—C9	1.513 (4)
N3—C4	1.461 (4)	C8—H8A	0.9700
N3—C5	1.464 (4)	C8—H8B	0.9700
C2—H2A	0.9600	C9—C10	1.377 (4)
C2—H2B	0.9600	C9—C14	1.387 (4)
C2—H2C	0.9600	C10—C11	1.395 (4)
C3—H3A	0.9600	C10—H10	0.9300
C3—H3B	0.9600	C11—C12	1.361 (4)
C3—H3C	0.9600	C11—H11	0.9300
C4—H4A	0.9600	C12—C13	1.372 (4)
C4—H4B	0.9600	C12—H12	0.9300
C4—H4C	0.9600	C13—C14	1.385 (4)
C5—C6	1.518 (4)	C13—H13	0.9300
C5—H5A	0.9700	C14—H14	0.9300
			
C1—N1—C8	122.4 (2)	C7—C6—C5	107.8 (2)
C1—N1—C7	122.5 (2)	C7—C6—H6A	110.1
C8—N1—C7	115.1 (2)	C5—C6—H6A	110.1
C1—N2—C2	121.8 (3)	C7—C6—H6B	110.1
C1—N2—C3	121.7 (2)	C5—C6—H6B	110.1
C2—N2—C3	116.3 (3)	H6A—C6—H6B	108.5
C1—N3—C4	120.6 (3)	N1—C7—C6	111.4 (2)
C1—N3—C5	115.9 (2)	N1—C7—H7A	109.3
C4—N3—C5	117.4 (2)	C6—C7—H7A	109.3
N1—C1—N3	118.9 (3)	N1—C7—H7B	109.3
N1—C1—N2	121.2 (3)	C6—C7—H7B	109.3
N3—C1—N2	119.8 (3)	H7A—C7—H7B	108.0
N2—C2—H2A	109.5	N1—C8—C9	113.7 (2)
N2—C2—H2B	109.5	N1—C8—H8A	108.8
H2A—C2—H2B	109.5	C9—C8—H8A	108.8
N2—C2—H2C	109.5	N1—C8—H8B	108.8
H2A—C2—H2C	109.5	C9—C8—H8B	108.8
H2B—C2—H2C	109.5	H8A—C8—H8B	107.7
N2—C3—H3A	109.5	C10—C9—C14	118.9 (3)
N2—C3—H3B	109.5	C10—C9—C8	120.1 (3)
H3A—C3—H3B	109.5	C14—C9—C8	120.9 (3)
N2—C3—H3C	109.5	C9—C10—C11	120.6 (3)
H3A—C3—H3C	109.5	C9—C10—H10	119.7
H3B—C3—H3C	109.5	C11—C10—H10	119.7
N3—C4—H4A	109.5	C12—C11—C10	119.9 (3)
N3—C4—H4B	109.5	C12—C11—H11	120.1
H4A—C4—H4B	109.5	C10—C11—H11	120.1
N3—C4—H4C	109.5	C11—C12—C13	120.3 (3)
H4A—C4—H4C	109.5	C11—C12—H12	119.9
H4B—C4—H4C	109.5	C13—C12—H12	119.9
N3—C5—C6	107.6 (2)	C12—C13—C14	120.3 (3)
N3—C5—H5A	110.2	C12—C13—H13	119.9
C6—C5—H5A	110.2	C14—C13—H13	119.9
N3—C5—H5B	110.2	C13—C14—C9	120.1 (3)
C6—C5—H5B	110.2	C13—C14—H14	120.0
H5A—C5—H5B	108.5	C9—C14—H14	120.0
			
C8—N1—C1—N3	147.7 (3)	C1—N1—C7—C6	14.4 (4)
C7—N1—C1—N3	−30.0 (4)	C8—N1—C7—C6	−163.5 (3)
C8—N1—C1—N2	−28.8 (4)	C5—C6—C7—N1	31.7 (3)
C7—N1—C1—N2	153.4 (3)	C1—N1—C8—C9	−40.3 (4)
C4—N3—C1—N1	145.8 (3)	C7—N1—C8—C9	137.6 (3)
C5—N3—C1—N1	−6.1 (3)	N1—C8—C9—C10	142.5 (3)
C4—N3—C1—N2	−37.6 (4)	N1—C8—C9—C14	−41.0 (4)
C5—N3—C1—N2	170.5 (2)	C14—C9—C10—C11	0.7 (5)
C2—N2—C1—N1	−39.7 (4)	C8—C9—C10—C11	177.3 (3)
C3—N2—C1—N1	135.0 (3)	C9—C10—C11—C12	1.6 (5)
C2—N2—C1—N3	143.8 (3)	C10—C11—C12—C13	−2.1 (5)
C3—N2—C1—N3	−41.6 (4)	C11—C12—C13—C14	0.4 (5)
C1—N3—C5—C6	52.6 (3)	C12—C13—C14—C9	1.8 (5)
C4—N3—C5—C6	−100.2 (3)	C10—C9—C14—C13	−2.4 (5)
N3—C5—C6—C7	−63.5 (3)	C8—C9—C14—C13	−178.9 (3)
